# Global Analysis of Microbiota Signatures in Four Major Types of Gastrointestinal Cancer

**DOI:** 10.3389/fonc.2021.685641

**Published:** 2021-08-05

**Authors:** Jihan Wang, Yangyang Wang, Zhenzhen Li, Xiaoguang Gao, Dageng Huang

**Affiliations:** ^1^Institute of Medical Research, Northwestern Polytechnical University, Xi’an, China; ^2^School of Electronics and Information, Northwestern Polytechnical University, Xi’an, China; ^3^Department of Spine Surgery, Honghui Hospital, Xi’an Jiaotong University, Xi’an, China

**Keywords:** microbiota signature, gastrointestinal cancer, TCMA, TCGA, biomarker

## Abstract

The gut microbiota has been previously linked with tumorigenesis and gastrointestinal cancer progression; however, intra-tumor microbiota analysis has just emerged and deserves increasing attention. Based on the public databases of The Cancer Microbiome Atlas (TCMA) and The Cancer Genome Atlas (TCGA), this study identified the tissue/organ microbial signatures generated from 443 biosamples of four major gastrointestinal cancer types, including esophageal carcinoma (ESCA), which further includes esophageal adenocarcinoma (EAD) and esophageal squamous cell carcinoma (ESCC), stomach adenocarcinoma (STAD), colon adenocarcinoma (COAD), and rectum adenocarcinoma (READ). According to partial least squares discrimination analysis (PLS-DA), the profile differences in microbial communities between the tumor and normal samples were not particularly noticeable across the four cancer cohorts, whereas paired comparison analyses revealed several specific differences in bacteria between tumor and normal samples in the EAD, STAD, and COAD samples. The taxa classified from the phylum to genus level revealed a trend of distinguishable microbial profiles between upper and lower gastrointestinal tumors. The Bacteroidetes/Firmicutes ratio in lower gastrointestinal tract tumors was nearly three times that in upper gastrointestinal tract tumors. We also determined the relative tissue/organ-prevalent microbes for each of the four cohorts at the order and genus levels. Microbe *Alistipes*, *Blautia*, *Pasteurellales*, and *Porphyromonas* compositions were correlated with the clinical characteristics of patients with gastrointestinal cancer, particularly colorectal cancer. Taken together, our findings indicate that microbial profiles shift across different gastrointestinal cancer types and that microbial colonization is highly site-specific. Composition of specific microbes can be indicative of cancer stage or disease progression. Overall, this study indicates that the microbial community and abundance in human tissues can be determined using publicly available data, and provides a new perspective for intra-tissue/organ microbiota research.

## Introduction

Gastrointestinal (GI) cancers are responsible for one-third of cancer mortality (1). According to statistics, an estimated 4.8 million new cases and 3.4 million related deaths of GI cancers occurred in 2018, accounting for 26% of all cancer incidence and 35% of cancer-related deaths. Approximately 8 in 100 men and 4 in 100 women are estimated to develop GI cancer before the age of 75, and more than half of new cases and related deaths occurred in Asia (2). Microorganisms, including bacteria, fungi, and viruses, have been described in terms of health and disease status (3–5). Nearly one-fifth of all cancers worldwide are linked with viral, parasitic, or bacterial infections; for instance, hepatitis B virus, human papillomavirus, and *Helicobacter pylori* are associated with hepatocellular carcinoma, cervical cancer, and stomach cancer, respectively (6). The human GI tract, in particular, harbors thousands of microbes. For example, the intestines have a dense community of approximately 10^13^ (7) microbes, whereas the stomach has the lowest microbial abundance due to its extreme acidity. These large numbers of microbial species constitute the microbiota, which refers to an ecological community of microbes that is found within a specific environment. The microbiota interacts with different types of host cells to modulate the organ microenvironment and to regulate physiological functions (8). Pathophysiological changes in cells and alterations in the microbial signature could have a significant impact on tumor occurrence and progression (9, 10), especially as microbial colonization is highly site-specific, allowing them to modulate the tumor microenvironment. The bacterial effects on cancer progression are related to the time and location of colonization (11), as well as on other pathogenic factors. In GI cancers, the microbiota has been recognized to be related to chemotherapy, radiotherapy, and immunotherapy efficacy (7, 8, 12, 13), indicating that the intestinal microbiota is a novel target to improve anti-tumor treatment (13). The presence of microbes within tumors and adjacent normal tissues may indicate disease progression and their potential roles in cancer pathogenesis (14–16). Understanding the alterations in the microbial community and abundance in GI organs thus aids in the study of GI cancer diagnosis and therapy.

Currently, the study of microbiota in life sciences has been greatly enhanced by advances in sequencing technology, accompanied by the application of multi-omics analysis (17). Intra-tumor microbiota analysis has recently emerged and has gradually increased in cancer studies (18–21). The Cancer Genome Atlas (TCGA) is a landmark cancer genomics program that sequenced and molecularly characterized 20,000 primary cancer and matched normal samples for 33 cancer types (22), which provides significant assistance in cataloguing and exploring cancer-causing genomic alterations and establishing a comprehensive “atlas” of cancer genomic signatures. Furthermore, the TCGA platform incorporates highly standardized clinical information regarding samples. Notably, the sequencing data in the TCGA offers a unique opportunity to study tissue/organ-related microbiota. Bioinformatics approaches authenticate microbiome research in the context of cancer-associated pathogenesis by using human sequencing data to characterize microbial profiles (bacterial, viral, or fungi). The Cancer Microbiome Atlas (TCMA, https://tcma.pratt.duke.edu) is a collection of curated, decontaminated microbial compositions of oropharyngeal, esophageal, GI, and colorectal tissues (23) based on samples from the TCGA database. At different taxonomic levels, the bacterial signatures of tumor and normal samples from patients with head and neck squamous cell carcinoma (HNSC), esophageal carcinoma (ESCA), stomach adenocarcinoma (STAD), colon adenocarcinoma (COAD), and rectum adenocarcinoma (READ) can be identified from TCMA, providing an excellent and powerful resource for studying the microbiome of GI cancers.

The objective of the current study was to investigate the microbiota profile in four major types of GI cancers, including ESCA, STAD, COAD, and READ. To identify the differences in microbial abundance between matched tumor-normal groups, the global microbiome signature at different taxonomic levels in both tumor and normal samples was analyzed. We also characterized the microbiome signature and identified relatively organ-prevalent microbes for each of the four GI cancer types to gain a better understanding of their similarities and heterogeneity based on their microbiome signatures. Furthermore, the correlation between specific candidate microbes and clinical variables of GI cancers was investigated by combining the TCMA microbial profile with the phenotype and survival data from TCGA. We believe that this is the first study to focus on the microbial composition of internal organs and their associations with four GI cancer types, which will provide evidence and a theoretical foundation for studying microbiome–host interactions and the role of the microbiome in digestive system malignant diseases.

## Materials and Methods

### Data Acquisition From TCMA and TCGA

**Figure 1:**
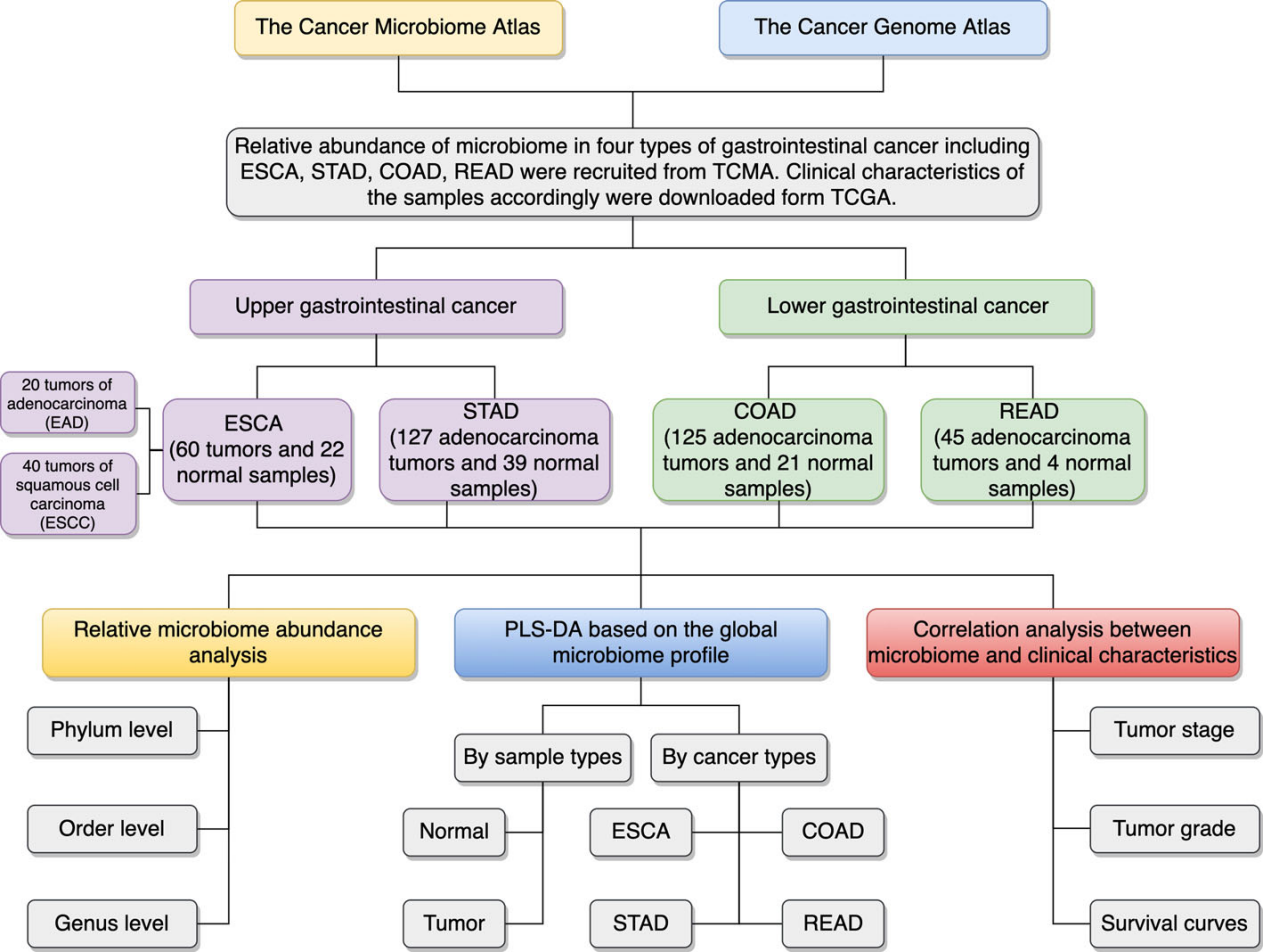
Schematic overview of the study design and workflow. TCMA, The Cancer Microbiome Atlas; TCGA, The Cancer Genome Atlas; ESCA, esophageal carcinoma; EAD, esophageal adenocarcinoma; ESCC, esophageal squamous cell carcinoma; STAD, stomach adenocarcinoma; COAD, colon adenocarcinoma; READ, rectum adenocarcinoma; PLS-DA, partial least squares discrimination analysis.

The microbial abundance profiles at different taxonomic levels were obtained from TCMA database for GI cancers including ESCA [specifically, including 20 tumors of esophageal adenocarcinoma (EAD), 40 tumors of esophageal squamous cell carcinoma (ESCC), and 22 normal samples], STAD (127 tumors of stomach adenocarcinoma and 39 normal samples), COAD (125 tumors of colon adenocarcinoma and 21 normal samples), and READ (45 tumors of rectum adenocarcinoma and 4 normal samples). TCGA includes biospecimens and the associated clinical information from human subjects under informed consent and authorization of local institutional review boards. We extracted the information about age, sex, race, tumor stage, and neoplasm histologic grade from the phenotype files, and about survival status and survival time from the survival files of TCGA data, we then integrated the microbial abundance profiles from TCMA and the clinical characteristics from TCGA for all the samples for further analysis. **Figure 1** depicts the study design and workflow. The clinical characteristics of the four types of GI tumors are summarized in **Table 1**.

**Table 1:** Clinical characteristics of the four types of gastrointestinal tumors in this study (derived from The Cancer Microbiome Atlas and The Cancer Genome Atlas databases).

Clinical characteristics	ESCA			STAD		COAD		READ	
	Tumor (EAD)	Tumor (ESCC)	Normal	Tumor	Normal	Tumor	Normal	Tumor	Normal
Age [median (min–max)]	72.75 (47–86)	59.45 (36–90)	77 (51–90)	68 (39–91)	72 (43–88)	69 (31–90)	68 (47–90)	66 (33–89)	58.5 (49–67)
Gender [number (%)]									
Male	15 (75.00)	36 (90.00)	15 (68.18)	82 (64.57)	24 (61.54)	61 (48.80)	10 (47.62)	22 (48.89)	1 (25.00)
Female	5 (25.00)	4 (10.00)	7 (31.2)	45 (33.86)	15 (38.46)	64 (51.20)	11 (52.38)	23 (51.11)	3 (75.00)
Race [number (%)]									
White	18	14	21 (95.45)	70 (55.12)	24 (61.54)	42 (33.60)	5 (23.81)	5 (11.11)	0 (0.00)
Black	0 (0.00)	2	0 (0.00)	2 (1.57)	1 (2.56)	3 (2.40)	0 (0.00)	1 (2.22)	1 (25.00)
Asian	1	24	0 (0.00)	14 (11.02)	1 (2.56)	21 (16.80)	0 (0.00)	0 (0.00)	0 (0.00)
Not reported	1	0 (0.00)	1 (4.55)	41 (32.28)	13 (33.33)	59 (47.20)	16 (76.19)	39 (86.67)	3 (75.00)
Tumor stage [number (%)]									
Stage I	6 (30.00)	2 (5.00)		21 (16.54)		27 (21.60)		10 (22.22)	
Stage II	4 (20.00)	26 (65.00)		40 (31.50)		47 (37.60)		17 (37.78)	
Stage III	6 (30.00)	9 (22.50)		33 (25.98)		35 (28.00)		12 (26.67)	
Stage IV	1 (5.00)	1 (2.50)		18 (14.17)		14 (11.20)		6 (13.33)	
Not reported	3 (15.00)	2 (5.00)		15 (11.81)		2 (1.60)		0 (0.00)	
Neoplasm histologic grade [number (%)]									
Grade X	11 (55.00)	4 (10.00)		0 (0.00)		Not applicable		Not applicable	
Grade 1	1 (5.00)	4 (10.00)		4 (3.15)					
Grade 2	4 (2.00)	23 (57.50)		43 (33.86)					
Grade 3	4 (2.00)	9 (22.50)		80 (62.99)					
Total [number (%)]	20 (100)	40 (100)	22 (100)	127 (100)	39 (100)	125 (100)	21 (100)	45 (100)	4 (100)

ESCA, esophageal carcinoma; EAD, esophageal adenocarcinoma; ESCC, esophageal squamous cell carcinoma; STAD, stomach adenocarcinoma; COAD, colon adenocarcinoma; READ, rectum adenocarcinoma.

### Analysis of Global Microbiota Profiles at Various Taxonomic Levels

The global microbial abundance profiles at the phylum, order, and genus taxonomic levels were downloaded from the TCMA database. We performed partial least squares discrimination analysis (PLS-DA) to investigate the overall differences in microbiota profiles between the tumor and normal groups for each cancer type, as well as in the tumor samples among the four major GI cancer types.

### Microbial Abundance Calculation and Analysis at Different Taxonomic Levels

Microbial abundance (percentage abundance) was calculated at the phylum, order, and genus taxonomic levels, and the microbiota profiles of the top five most abundant microbes at the phylum level and the top 10 most abundant microbes at the order/genus levels were summarized for further study. We used the paired two-tailed Student’s t-test to compare microbial abundance in the tumor versus paired-normal samples, with *P* < 0.05 representing statistical significance. To examine the similarities and heterogeneities among the four types of GI cancer, a Venn diagram was drawn (http://bioinformatics.psb.ugent.be/webtools/Venn/) and bi-cluster analysis (using the “pheatmap” package in R version 4.0.2) based on microbiota profiles was performed.

### Correlation Analysis of Microbial Abundance and Clinical Characteristics

Pearson correlation was performed in R version 4.0.2 using the cor.test () algorithm to analyze the correlation of specific microbial abundance and clinical characteristics, including tumor stage and histologic sample grade (*P* < 0.05). The Kaplan–Meier model from the survival and survminer packages in R version 4.0.2 was used for survival analysis based on microbial abundance. The microbial abundance values were divided into high (high) and low (low) groups based on median values, with *P* < 0.05 representing statistical significance.

## Results

### Microbiota Profile Landscape of GI Cancers

**Figure 2:**
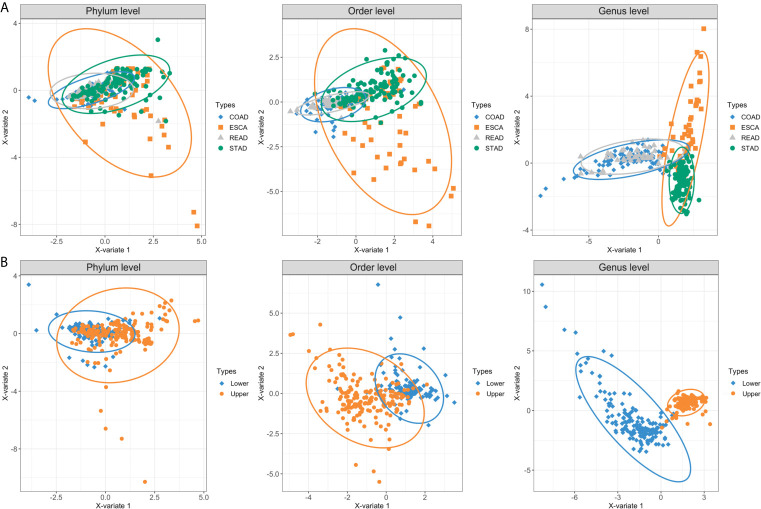
PLS-DA plots based on the microbial profile of four GI cancer types. **(A)** PLS-DA plots of four cancer cohorts including ESCA, STAD, COAD, and READ at the phylum, order, and genus levels. **(B)** ESCA and STAD were further collected as upper GI tumors, whereas COAD and READ were collected as lower GI tumors. The light ellipse in each group represents the 95% confidence interval (CI) of the score calculated from the corresponding group. PLS-DA, partial least squares discrimination analysis; ESCA, esophageal carcinoma; STAD, stomach adenocarcinoma; COAD, colon adenocarcinoma; READ, rectum adenocarcinoma.

Overall, we collected and integrated the microbiota profile and clinical characteristics of 443 GI cancer samples (including 357 tumor samples and 86 normal samples) from four cohorts. In total, 11 phyla, 38 orders, and 221 genera of microbial taxa were extracted from each sample from the TCMA database. First, we used a PLS-DA plot to compare the microbiota profile landscapes of tumor and normal samples from the same organ. The microbial profile could not well distinguish the sample type (tumor or normal) at the phylum, order, or genus levels, as shown in **Supplementary Figure 1**. We then focused on the hypothesis that microbial composition signatures are associated with different organs/tissues of GI tumors. As shown in **Figure 2**, we discovered that microbes have a highly organ-dependent signature. For example, the global microbiota profile of STAD is closer to that of ESCA (including EAD and ESCC) at the genus level, and samples from READ were nearly overlapped with the COAD group (**Figure 2A**). Furthermore, by combining ESCA and STAD samples as one type (upper GI tumor), and COAD and READ samples as another type (lower GI tumor), a clear distinction was found between upper and lower GI tumors, with the taxonomic rank ranging from the phylum to genus level (**Figure 2B**).

### Microbiota Taxonomic Composition in GI Cancers

**Figure 3:**
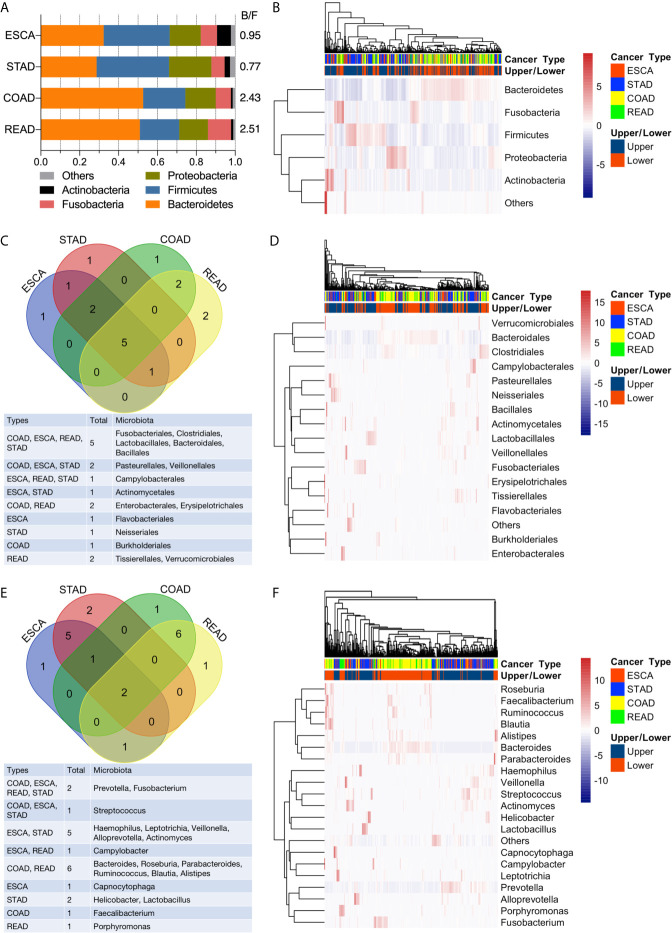
Analysis of microbial abundance in GI cancers. **(A)** The top five most abundant microbial phyla in the four cancer types; the B/F ratio represents the Bacteroidetes/Firmicutes ratio. **(B)** Bi-clustering heatmap based on the top five abundant microbial profiles at the phylum level. The horizontal axis represents the samples, and the vertical axis represents the microbial taxa. **(C)** Venn diagram of the top 10 abundant microbial compositions at the order level. **(D)** Bi-clustering heatmap based on the top 10 abundant microbial profiles at the order level. **(E)** Venn diagram of the top 10 abundant microbial compositions at the genus level. **(F)** Bi-clustering heatmap based on the top 10 abundant microbial profiles at the genus level.

*Bacteroidetes*, *Firmicutes*, *Proteobacteria*, *Fusobacteria*, and *Actinobacteria* dominated the top 5 abundant taxa at the phylum level (**Figure 3A, B**). In the ESCA (including EAD and ESCC) tumor samples, *Bacteroidetes* (0.32), *Firmicutes* (0.34), *Proteobacteria* (0.16), *Fusobacteria* (0.08), and *Actinobacteria* (0.07) constituted nearly 97% of the microbiota phyla, the composition of which was similar to that of STAD samples: *Bacteroidetes* (0.29), *Firmicutes* (0.37), *Proteobacteria* (0.22), *Fusobacteria* (0.07), and *Actinobacteria* (0.03). Samples from the lower GI tumor had significantly higher levels of *Bacteroidetes* (0.53 for COAD, 0.51 for READ) and lower levels of *Firmicutes* (0.22 for COAD, 0.20 for READ). Thus, the lower GI tumor samples had a clearly higher *Bacteroidetes*/*Firmicutes* ratio compared to the upper GI tumor group (**Figure 3A**). However, the bi-clustering heatmap at the phylum level could not distinguish between the four cancer types (**Figure 3B**). The top 10 abundant microbiota taxa in each of the four cancer types were calculated and analyzed at the order and genus levels (**Figures 3C–F**). At the order level, half (5/10) of the most abundant microbiota were shared by all four cancer types (**Figure 3C**). Compared to the bi-clustering result at the phylum level, we observed a more obvious clustering trend at the order level, with ESCA clustering closer to STAD samples and READ clustering closer to the COAD group (**Figure 3D**). Furthermore, the difference in microbiome heterogeneity between the upper and lower GI tumors was more pronounced at the genus level. The composition of the top 10 abundant genera differed between the upper and lower GI tumors, as shown in **Figure 3E**. For instance, only two genera were shared by all four cancer types, the samples in the ESCA and STAD groups had five shared-genera, whereas the samples in the COAD and READ groups had six shared-genera. Furthermore, the organ-prevalent genera were identified relatively. The microbial *Capnocytophaga* presence ratios in the four cancer types were 18/60 (ESCA), 18/127 (STAD), 0/125 (COAD), and 0/45 (READ), respectively. *Helicobacter* genus had existence ratios of 5/60 (ESCA), 42/127 (STAD), 0/125 (COAD), and 0/45 (READ), respectively. The existence ratios for the *Faecalibacterium* genus were 0/60 (ESCA), 0/127 (STAD), 35/125 (COAD), and 11/45 (READ), respectively. *Porphyromonas* was found in nearly half of the READ samples (19/45), but less in the other three cancer types (6/60 for ESCA, 25/127 for STAD, 17/125 for COAD). Through the bi-clustering heatmap, we observed a relatively distinguishable pattern between upper GI and lower GI tumors based on their microbial profiles at the genus level (**Figure 3F**).

**Figure 4:**
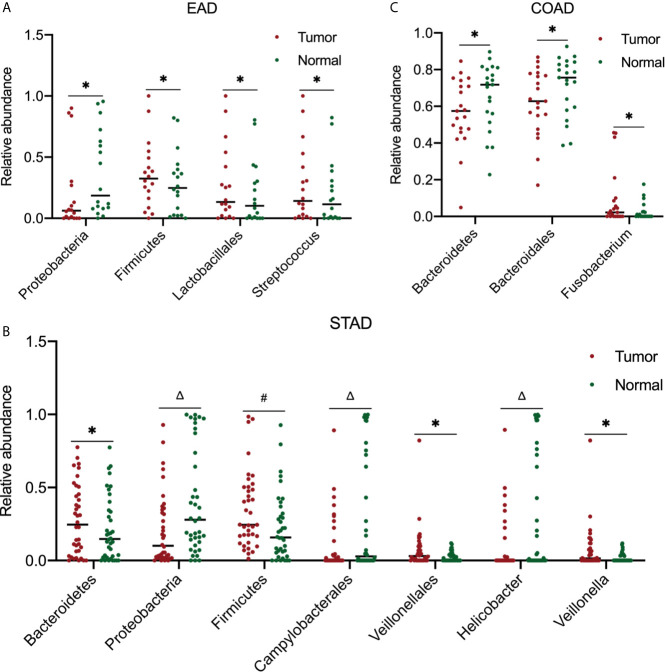
Analysis of microbiota abundance differences between tumor and paired normal samples. In EAD **(A)**, STAD **(B)**, and COAD **(C)**, there were 18, 38, and 21 paired tumor-normal samples, respectively. For significance analysis, a paired two-tailed Student’s t-test was used. *P < 0.05, ^#^ *P* < 0.01, ^Δ^ *P* < 0.001 compared to the normal group. The differences between tumor and paired normal samples were not significant in ESCC and READ samples (data not shown). EAD, esophageal adenocarcinoma; ESCC, esophageal squamous cell carcinoma; STAD, stomach adenocarcinoma; COAD, colon adenocarcinoma; READ, rectum adenocarcinoma.

Furthermore, the most abundant microbial composition was used to analyze the differences in abundance between tumor and normal samples in the same organ. To obtain more accurate results, we used a two-tailed Student’s t-test to compare the tumor and strictly paired normal samples for each cancer type. For ESCA, there were 18 and 4 paired tumor/normal samples for EAD and ESCC, respectively; for STAD, COAD, and READ, there were 38, 21, and 4 paired tumor/normal samples, respectively. **Figure 4** summarizes the statistically significant outcomes for the EAD, STAD, and COAD groups. There were no statistically significant differences between the tumor and paired normal samples for the ESCC and READ groups, possibly because of the small sample size of the two groups.

### Microbiota Associated With Clinical Characteristics and Survival Status in GI Cancers

**Figure 5:**
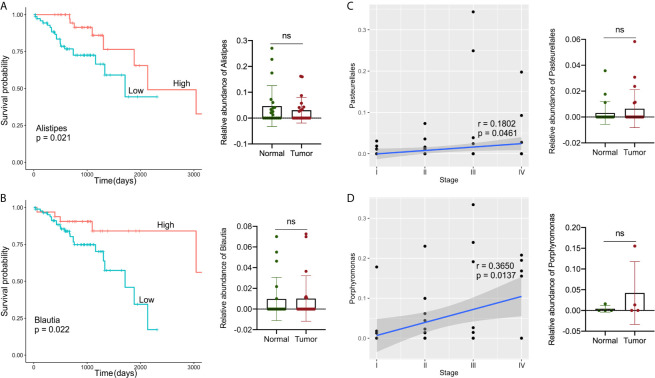
Analysis of correlations between specific microbes and the clinical characteristics or survival status in GI cancers. **(A, B)** Kaplan–Meier survival curves based on microbial abundances of Alistipes **(A)** and Blautia **(B)**, and calculation of their abundance in COAD tumor and paired normal samples. **(C)** Correlation of Pasteurellales abundance with the tumor stage, and calculation of Pasteurellales abundance in COAD tumor and paired normal samples. **(D)** Correlation of *Porphyromonas* abundance with tumor stage, and calculation of *Porphyromonas* abundance in READ tumor and paired normal samples. COAD, colon adenocarcinoma; READ, rectum adenocarcinoma.

TCGA collects comprehensive clinicopathological annotation data, allowing researchers to investigate disease-related factors in cancer. After integrating the microbial abundance profile from TCMA and the clinical characteristics from TCGA, we investigated whether there were specific candidate microbial taxa that correlated with the clinical characteristics or survival status of GI cancers, as specific microbes have potential value as disease-related biomarkers. We discovered that the relative abundance of several microbial compositions was related to the overall survival rate or stage status in GI cancer patients, especially for COAD and READ (**Figure 5**). The high abundances of *Alistipes* and *Blautia* in tumor samples were correlated with better survival probability in patients with COAD (*P* < 0.05), and the relative levels of *Alistipes* and *Blautia* in the tumor were slightly decreased compared to their paired normal samples, but no significant difference was found (**Figures 5A, B**). Furthermore, the relative abundance of *Pasteurellales* was slightly increased (with no significant difference) in tumors compared with that in normal tissues and was positively correlated with COAD tumor stage (*P* < 0.05, **Figure 5C**). In READ, the relative abundance of *Porphyromonas* in tumors was elevated (with no significant difference) when compared with paired normal samples and was positively correlated with tumor stage (*P* < 0.05, **Figure 5D**).

## Discussion

Numerous studies have identified links between the microbiota and tumorigenesis and progression in various cancer types (24, 25). Until now, the majority of related studies have concentrated primarily on the role of the gut microbiota (GM) in disease. Several studies have recently characterized the profile of tissue-resident microbiota in various cancers (6, 11, 18, 19, 26). Identification of microbial communities and abundance derived from human tissues and organs was significantly assisted by publicly available genome sequencing data in the TCGA database.

Herein, we described the global microbial signature associated with four major types of GI cancers by conducting a comprehensive analysis of the bacterial taxa in the TCMA. Overall, across the four cancer cohorts, the PLS-DA profile differences in the microbial communities between tumor and normal samples were not particularly noticeable. Despite this, the abundance of specific bacteria between strict paired tumor-normal samples were different. In the STAD cohort and EAD samples from the ESCA cohort, the relative abundance of *Firmicutes* in tumor samples was increased, whereas that of *Proteobacteria* was decreased significantly compared to that in normal samples. The link between *Helicobacter pylori* infection and gastric cancer has been well established. Noteworthy consistent with other studies (11, 27), the abundance of *Helicobacter* was higher, whereas that of *Veillonellales/Veillonella* was lower in the paired normal samples compared to tumor samples (38 paired of tumor/normal samples in this study) within the gastric cohort. In COAD, the tumor samples had significantly lower levels of *Bacteroidetes* and *Bacteroidales* and higher levels of *Fusobacterium* compared to their normal counterparts.

In our study, we observed distinct microbial profiles between the upper and lower GI tumors, as the taxa were classified from the phylum to genus level, whereas minor differences were found in the microbiota signature between ESCA and STAD in the upper GI tract and COAD and READ in the lower GI tract. As exploring tissue-resident microbiota profiles can help to identify predictive microbial biomarkers for a specific cancer type, we further concentrated on identifying and comparing the common and distinct microbial taxa in four GI cancer types. At the phylum level, *Bacteroidetes* and *Firmicutes* dominated the microbial composition in the ESCA and STAD cohorts of the upper GI tract, whereas *Bacteroidetes* dominated the lower GI tract samples of COAD and READ. At the order level, the four cancer cohorts shared half of the top 10 most detected microbial compositions. Furthermore, a trend of clustering was observed between ESCA and STAD, as well as between COAD and READ; the clustering phenomenon and differences in microbial profiles within groups were most visible at the genus level. According to our findings, only two common abundant microbial genera were detected in the four cancer types. Previous research has shown that the genera *Streptococcus*, *Lactobacillus*, *Veillonella*, and *Prevotella* predominate in the gastric microbiota (28), which is consistent with the current findings. The STAD group nearly overlapped the most abundant genus profile in ESCA samples. Several upper GI tract microbial genera (*Streptococci*, *Veillonella*, *Lactobacillus*) were reported in abundance in the microbial community coating the tongue (27), indicating that anatomically adjacent organs have relatively similar microbial signatures. The READ cohort had the most common abundant genera with the COAD cohort in the lower GI tract. Colorectal cancer (CRC) is closely correlated with dramatic changes in microbial composition, also known as dysbiosis (29, 30). Evidence for important roles of *Fusobacterium nucleatum*, *Escherichia coli*, and *Bacteroides fragilis* as specific strains associated with CRC is also emerging (31). CRC-associated microbiota profiles differ from those found in healthy subjects; the microbiota composition in colorectal cancer in our study was similar to that found in other studies (32, 33). We also identified tissue/organ-specific flora. For example, *Capnocytophaga* and *Helicobacter* were only found in the ESCA and STAD cohorts. It is known that *Helicobacter pylori* is a major etiological factor in the development of upper GI tract conditions (34), and its infection in the stomach is a risk factor for STAD prognosis (35). On the contrary, *Faecalibacterium* was found only in CRC samples.

Finally, we examined the relationship between candidate microbes and clinical variables in patients after combining the microbiome profile from TCMA and clinical characteristic information from TCGA for all samples, focusing on factors such as tumor stage, histologic grade, and overall survival status. In general, we discovered more microbial correlations with CRC clinical characteristics than with upper GI cancers. For example, the abundance of *Alistipes* and *Blautia* was moderately decreased in tumors compared to that in the paired normal samples, and their high level indicated a better survival probability in patients with COAD. The composition of *Pasteurellales* and *Porphyromonas* was related to the tumor stage status of COAD and READ, respectively. Recently, there has been contradictory evidence indicating the two-sided effects of *Alistipes* on health. *Alistipes* may confer protective effects against diseases such as liver fibrosis, colitis, and cardiovascular disease (36). Other studies have found *Alistipes* to be pathogenic in colorectal cancer (33, 36, 37), which contradicts the results of the current study and requires further clarification. In a study on mucosa-adherent microbiota, *Blautia* was found to be lower in patients with CRC than in healthy controls (38). Several studies (39, 40) have found high levels of *Porphyromonas* in colorectal cancer, which is consistent with our findings. The results indicate that a novel approach to microbial-based cancer discrimination and prognosis prediction may provide significant future value to patients.

Our study has some limitations; the small size of paired tumor-normal samples weakened the power of the comparison study, particularly in the READ cohort and the ESCC subgroup of ESCA. Further, the clinicopathological data in this study need to be supplemented and completed to obtain more comprehensive results regarding the relationship between GI cancers and the microbiota. Besides, the current study is more of an observational research, and interference study is essential and need to be conducted in the future to eliminate the false correlation drawing from bioinformatics data.

## Conclusion

In this study, we characterized the microbiota signatures of four major GI cancer types: ESCA (including EAD and ESCC), STAD, COAD, and READ. Taken together, our findings indicate that microbial profiles differ noticeably between upper and lower GI tissues/organs, and that microbial colonization is relatively site-specific. Several candidate microbial biomarkers can be predictive of tumor stage and cancer prognosis. This approach confirms the ability to identify the microbial community and abundance in human tissues based on publicly available genome sequencing data, helps to discover prognostic species, and enables systematic matched microbe-host multi-omic analyses, which provides a new perspective for intra-tissue/organ microbiota research and will help guide future studies of the microbiome’s role in human health and disease.

## Data Availability Statement

The original contributions presented in the study are included in the article/**Supplementary Material**. Further inquiries can be directed to the corresponding authors.

## Author Contributions

JW designed, performed the research, and co-wrote the manuscript. YW and XG contributed to data and statistical analysis. ZL co-wrote the manuscript. DH supervised the research. All authors contributed to the article and approved the submitted version.

## Funding

This work was supported by the Fundamental Research Funds for the Central Universities of Northwestern Polytechnical University (G2020KY0516), Shaanxi Provincial Key Research and Development Program (2020GXLH-Y-027, 2021SF-030), and the Innovative Talents Promotion Program (2020KJXX-023).

## Conflict of Interest

The authors declare that the research was conducted in the absence of any commercial or financial relationships that could be construed as a potential conflict of interest.

## Publisher’s Note

All claims expressed in this article are solely those of the authors and do not necessarily represent those of their affiliated organizations, or those of the publisher, the editors and the reviewers. Any product that may be evaluated in this article, or claim that may be made by its manufacturer, is not guaranteed or endorsed by the publisher.

## Supplementary Material

Supplementary Figure 1 PLS-DA plots based on microbial profile of tumor and normal samples from the same organ. PLS-DA plots at phylum, order, and genus levels of tumor and normal samples from ESCA **(A)**, STAD **(B)**, COAD **(C)**, and READ **(D)**.
