# Supplementary figures and images for: Global Analysis of Microbiota Signatures in Four Major Types of Gastrointestinal Cancer
Source: Front Oncol. 2021 Aug 5;11:685641. doi: 10.3389/fonc.2021.685641 (PMC8375155; doi:10.3389/fonc.2021.685641)

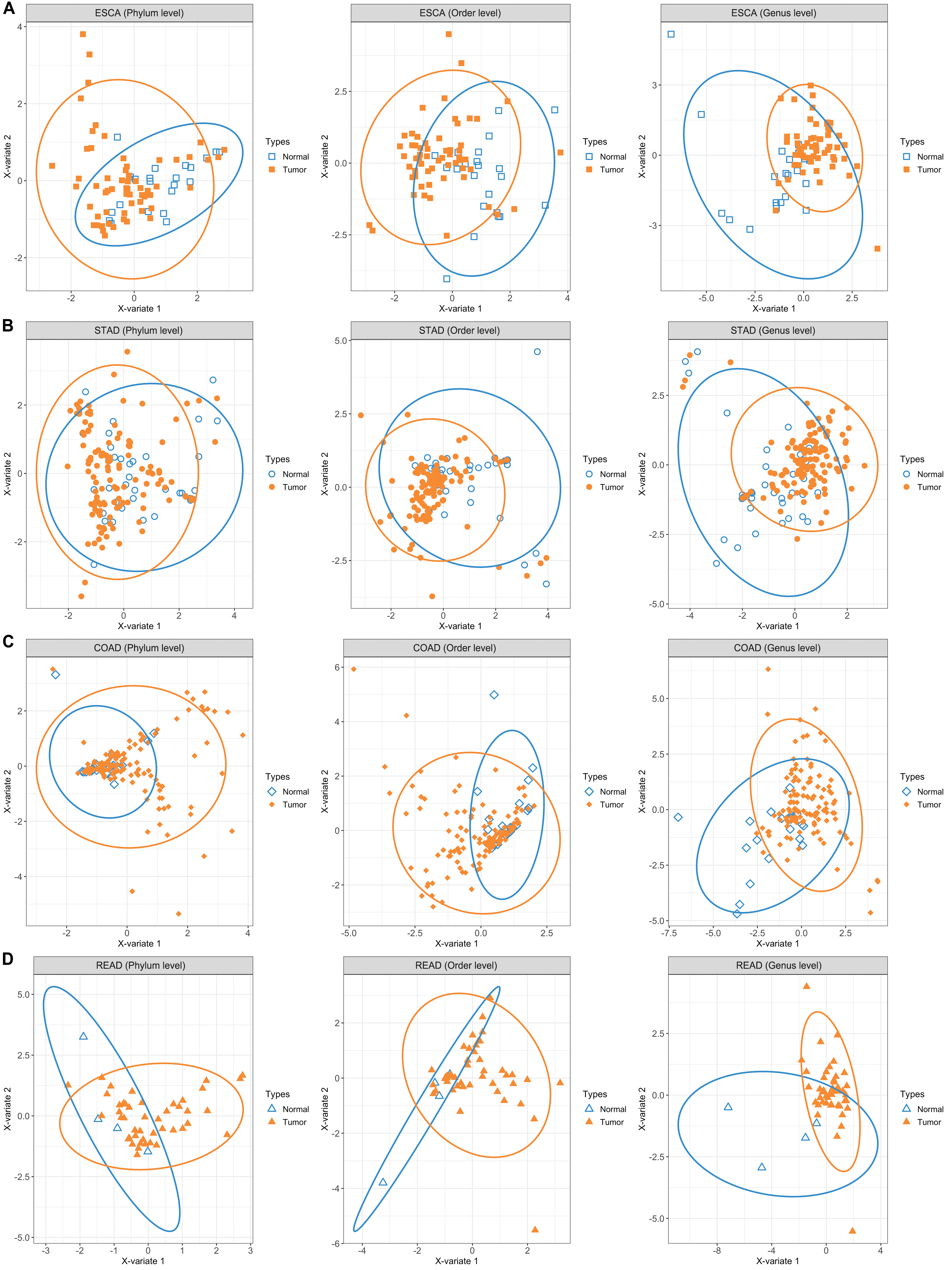

Supplement: Supplementary Figure 1 — PLS-DA plots based on microbial profile of tumor and normal samples from the same organ. PLS-DA plots at phylum, order, and genus levels of tumor and normal samples from ESCA (A), STAD (B), COAD (C), and READ (D). [file Image_1.tif]
